# Genetic, Phenotypic and Metabolic Diversity of Yeasts From Wheat Flag Leaves

**DOI:** 10.3389/fpls.2022.908628

**Published:** 2022-07-07

**Authors:** Linda Gouka, Caroline Vogels, Lars H. Hansen, Jos M. Raaijmakers, Viviane Cordovez

**Affiliations:** ^1^Department of Microbial Ecology, Netherlands Institute of Ecology (NIOO-KNAW), Wageningen, Netherlands; ^2^Department of Plant and Environmental Sciences, University of Copenhagen, Copenhagen, Denmark; ^3^Institute of Biology, Leiden, Netherlands

**Keywords:** yeast ecology, phyllosphere, (a)biotic stresses, antagonism, culturomics, functional characterization, biofilm formation, carbon utilization

## Abstract

The phyllosphere, the aboveground part of a plant, is a harsh environment with diverse abiotic and biotic stresses, including oscillating nutrient availability and temperature as well as exposure to UV radiation. Microbial colonization of this dynamic environment requires specific adaptive traits, including tolerance to fluctuating temperatures, the production of secondary metabolites and pigments to successfully compete with other microorganisms and to withstand abiotic stresses. Here, we isolated 175 yeasts, comprising 15 different genera, from the wheat flag leaf and characterized a selection of these for various adaptive traits such as substrate utilization, tolerance to different temperatures, biofilm formation, and antagonism toward the fungal leaf pathogen *Fusarium graminearum*. Collectively our results revealed that the wheat flag leaf is a rich resource of taxonomically and phenotypically diverse yeast genera that exhibit various traits that can contribute to survival in the harsh phyllosphere environment.

## Introduction

The phyllosphere is a reservoir of yet unknown microorganisms with intriguing interactions. Microorganisms colonizing the aboveground plant surfaces and tissues, including floral and vegetative parts, are referred to as the phyllosphere microbiome. The phyllosphere is considered to be a harsh environment due to the microbial exposure to limited nutrient sources, UV radiation, temperature oscillations and toxic compounds (Vorholt, 2012). Phyllosphere microorganisms display a wide range of adaptations and antagonistic activities, which are gaining interest for sustaining plant health (Legein et al., 2020; Kavamura et al., 2021). Among the phyllosphere-inhabiting microorganisms, yeasts are found to be abundant as endo- or epiphytes, reaching on average 10^3^–10^5^ colony forming units (CFU) per gram of leaf (Glushakova and Chernov, 2007). The majority of studies on the phyllosphere microbiome, so far, focuses on bacteria and filamentous fungi, especially plant pathogens. Currently, little is known about the diversity and ecological roles of phyllosphere yeasts (Kavamura et al., 2021).

Previous culture-dependent and independent approaches have shown a predominance of the Basidiomycete yeasts *Sporobolomyces*, *Cryptococcus* (often reclassified as *Papiliotrema*) and *Pseudozyma* (Nasanit et al., 2015a,c, 2016) in the phyllosphere of rice, corn, and sugarcane. Several biotic and abiotic factors impact the abundance and diversity of yeasts in the phyllosphere. These factors include plant genetics (e.g., plant species, genotype, and developmental stage) and environmental conditions such as UV radiation, moisture, geographic location and fungicides (Sapkota et al., 2015). For example, the abundance of Ascomycete yeasts, such as *Metschnikowia* and *Cryptococcus*, generally increased over time, particularly in nectar yielding flowers (Glushakova and Chernov, 2010). Additionally, an increase in nutrients found on damaged berries also increased the number of yeasts in the phyllosphere (Janakiev et al., 2019).

The phyllosphere is considered a resource-poor environment, thus microorganisms compete for nutrients and space. Nutrients leak from leaves or fruits into the environment, mainly consisting of carbohydrates such as fructose, glucose and sucrose, and also amino acids and methanol (Mercier and Lindow, 2000). Yeasts are known for their versatile substrate utilization (Żymańczyk-Duda et al., 2017), they can use different carbon sources (e.g., simple sugars, methanol, and methane) as well as amino acids and nitrogen sources (e.g., methylamine, ammonium salts and nitrate; Moliné et al., 2010; Chi et al., 2015; Shiraishi et al., 2015), allowing them to expand their ecological niche (Deak, 2006).

A key step to elucidate the ecological roles of phyllosphere yeasts lies in determining their ability to withstand harsh environmental conditions and their interactions with other phyllosphere members. A number of mechanisms have been proposed to facilitate the adaptations of yeasts to the phyllosphere environment. For example, the majority of phyllosphere yeasts are present in highly organized multicellular communities called biofilms (Váchová and Palková, 2018), which play a role in stress resilience. More specifically, biofilm formation has been implicated in resistance to fungicides and proposed as a physical barrier on plant surface injuries, preventing fungal hyphae from entering the plant tissue (Villa et al., 2017).

Currently, the majority of studies on environmental yeasts focusses on their biocontrol potential (Schisler et al., 2002a, 2015). Several studies have shown the ability of yeasts to inhibit plant pathogens and to protect against post-harvest diseases *via* the production of secondary metabolites, cell-wall-degrading enzymes, and so-called killer toxins (Freimoser et al., 2019). The mechanisms underlying these antagonistic activities have been identified only for a few species. For example, the production of pulcherriminic acid by *Metschnikowia pulcherrima* has been implicated in the growth inhibition of *Botrytis cinerea* (Sipiczki, 2020), whereas polymers (e.g., pullulan), volatiles (e.g., ethanol, phenylethanol and ethyl acetate) and secondary metabolites (e.g., aureobasidins) produced by *A. pullulans* have been shown to inhibit the growth of *Alternaria alternata* and *B. cinerea* (Contarino et al., 2019; Yalage Don et al., 2020; Di Francesco et al., 2021). For the majority of biocontrol yeasts, however, the mechanisms underlying the antagonistic activity are still unknown (Gore-Lloyd et al., 2019). Investigations of their lifestyles and adaptations to the phyllosphere environment will contribute to a better understanding of the interplay between yeasts and other phyllosphere members.

The present study aimed at investigating the taxonomic diversity of yeasts from wheat flag leaves, i.e., the last leaf before the ear emergence. The flag leaf can contribute up to 40% of the final photosynthetic capacity of wheat plants and therefore has a major impact on yield (Sylvester-Bradley et al., 1990). For a subset of taxonomically different flag leaf yeasts, we assessed their phenotypic and metabolic potential, including biofilm formation, substrate utilization spectrum, growth at different temperatures and antagonism toward the fungal pathogen *Fusarium graminearum*. Our results provide a first step toward characterizing the yeast diversity specifically found in wheat flag leaves and serve a foundation for further studies on the ecological roles of phyllosphere yeasts.

## Materials and Methods

### Isolation of Yeasts From the Wheat Flag Leaf

Yeasts were isolated from the flag leaves of wheat (Triticum aestivum) cultivar Elixer. Samples were collected during the spring of 2020 at Taastrup, Denmark (55°38′46″N 12°17′53″E) on a fungicide untreated field plot. Plants were sampled at the flowering stage (growth stage 61–69 according to Zadoks code (Zadoks et al., 1974)). Three different methods were used to isolate both epiphytic and endophytic yeasts. For the first method a washing solution (0.5% Tween80 and 0.9% NaCl) was added to a 15 ml-tube with 33 flag leaves. Samples were vortexed for 1 min, sonicated for 2 min at 45 kHz, and vortexed again for 1 min. Leaves were transferred to a new tube and blended. The blended-washed leaves were stored in 15% (v/v) glycerol. In the second method, the washing solution was centrifuged for 5 min at 5.000 rpm, the supernatant was removed and the pellet was stored in 3 ml of 15% glycerol (v/v). The third method consisted of blending leaves directly (without the washing step) and storing in 15% glycerol (v/v). All samples were stored at −80°C.

For the selective isolation of yeasts, glycerol samples were 10-fold serial diluted (10^−1^–10^−7^) with 0.9% NaCl and plated on different media. Suspensions were plated on Malt Extract Agar (MEA), Potato Dextrose Agar (PDA), Sabouraud Agar (SDA), 869 media supplemented with wheat flag leaf extract (Eevers et al., 2015) and Yeast Extract Peptone Dextrose (YEPD), with and without 10% lactic acid to favor yeast growth. Bacterial growth was prevented by adding 50 μl/ml chloramphenicol and 50 μl/ml tetracycline. Plates were incubated for 5–7 days at 25°C. At least two of each yeast colony morphologies were picked and streaked on fresh plates (at least twice) to obtain pure cultures. Isolates were grown in YEPD broth for 2–3 days and stored in 15% glycerol (v/v). Isolates which did not grow well in liquid were streaked on plates and, after 3–5 days, cells were directly resuspended in glycerol. All samples were stored at −80°C for long-term storage.

### Taxonomic Identification of Yeasts

All 175 yeast isolates were characterized by ITS rRNA gene sequencing. PCR amplifications were conducted using the primers ITS1 (5′-TCCGTAGGT GAACCTGCGG-3′) and ITS4 (5′-TCCTCCGCTT ATTGATATGC-3′), synthesized by Integrated DNA Technologies (IDT; White et al., 1990; Lane, 1991). Additionally, the selection of 51 isolates was also characterized by 28S rRNA gene sequencing (also known as D1/D2 sequencing) using the primers LR5 (5′-TCCTGAGG GAAACTTCG-3′) and LROR (5′-ACCCGCTG AACTTAAGC-3′). DNA template for colony PCR was obtained by disrupting the cells by heating in a microwave at 600 W for 60 s, followed by vortexing and again microwaving at 600 W for 60 s in sterile demineralized-water. Samples were centrifuged at 5.000 rpm for 5 min and an aliquot of 3 μl was used as DNA template. The reaction mixture contained Go Taq G2 Hot Start Green Master Mix 2×, 0.4 μm for both primers, DNA template and nuclease-free water. The PCR conditions in the Thermal cycler were: 95°C for 2 min, followed by 30 cycles of 95°C for 1 min, 55°C for 30 s, and 72°C for 1 min 30 s, with a final extension at 72°C for 5 min and stored at 12°C. The presence of the PCR products was verified on a 1% agarose gel in TBE buffer. PCR products were purified *via* ethanol precipitation or using a PCR purification kit (Zymo Research fungal/bacterial DNA Miniprep Kit). Samples were sequenced at BaseClear (Leiden, Netherlands) or Macrogen Inc. (Amsterdam, Netherlands) using the primer ITS1 and LR5. Taxonomic characterization was based on partial internal transcribed spacers (ITS) and the D1/D2 domains of the large subunit of 26S rRNA gene sequences using the NCBI BLAST database.^1^ For the phylogenetic analysis, sequences were aligned using the MUSCLE algorithm in the MEGA software (version 7.0) and a phylogenetic tree was constructed using Maximum Likelihood method and Tamura-Nei model with a bootstrap of 1.000 replications (Kumar et al., 2016). The tree was visualized using iTOL (version 6). ITS and D1/D2 sequences are deposited at European Nucleotide Archive (ENA) under the project number PRJEB51687.

### Culture Conditions

For all *in vitro* assays, the fungal isolate *F. graminearum* strain 8/1 (Miedaner et al., 2000) was grown on Potato Dextrose Agar (PDA, pH 7) plates for 5–7 days at 25°C. Yeast cultures were started from glycerol stocks. Isolates were plated on PDA plates and incubated at 25°C for 5–10 days. For all *in vitro* assays, a loop of the yeast cells was collected and inoculated in 0.9% NaCl. Cells were washed twice by centrifugation at 5.000 rpm for 5 min. An initial cell density (OD_600nm_) of 0.1 was used for all experiments except the Biolog plate, where an OD_600nm_ of 0.01 was used. All assays were performed at 25°C, which was the temperature used for the isolation of yeasts, unless stated otherwise.

### Metabolic Fingerprinting

The Biolog EcoPlate^™^ system (Biolog Inc., Hayward, CA, United States of America) were used to analyze differential utilization of carbon sources and thereby providing a metabolic fingerprint for each individual strain. Every plate contains 31 different carbon sources grouped into six categories (including amino acids, amines, carbohydrates, carboxylic acids miscellaneous, and polymer compounds) and a water control in three replicates. Yeast cells, described above, were adjusted to OD_600nm_ 0.01 and 100 μl per well was inoculated into the Biolog EcoPlate^™^. Plates were incubated at 25°C for 10 days at 180 rpm. Metabolism of specific substrates, and consequently growth of the cultures, resulted in a change in the tetrazolium dye. Cell density was measured after 2, 4, 7, and 10 days using a microtiter plate reader (OD_590nm_), minus values were adjusted to zero. The average well color development (AWCD) was calculated by dividing the total of all values (excluding the water control) by 93. Additionally, the AWCD was calculated for each substrate group. MetaboAnalyst (Version 5.0) was used to visualize the 10 day-inoculation data. A heatmap was created following a Euclidean distance measure and a ward clustering method (Xia et al., 2009).

### Growth at Different Temperatures

Yeast cells were collected as described above. The cell density was adjusted to OD_600nm_ 0.1 and 20 μl were added to 180 μl PDB pH 7 in a round-bottom microtiter 96-wells plate. Plates were sealed with plastic wrap and incubated at different temperatures, ranging from 4, 10, 25 to 37°C, at 200 rpm for 7 days. Cell density was measured after 2, 4, and 7 days using a microtiter plate reader (OD_600nm_).

### *In vitro* Biofilm Formation

Yeast cells were collected from plates and OD_600nm_ was adjusted to 0.1 as described above. A total of 20 μl of this suspension was added to 180 μl PDB in a flat-bottom microtiter plate. Plate was sealed and incubated statically in the dark for 3–4 days at 25°C. After incubation, cells were stained by adding 10 μl of 0.1% crystal violet to each well of the microtiter plate. Plate was incubated at room temperature for 15 min. Cells were washed three times with demineralized-water to remove platonic cells. After that, cells in biofilm were dissolved in 200 μl of 96% ethanol and incubated for 5 min. Cell density was measured using a microtiter plate reader at OD_600nm_.

### Antifungal Activity *via* Agar-Diffusible and Volatile Compounds

The effect of yeast diffusible compounds on the growth of the fungal pathogen *F. graminearum* was tested using *in vitro* dual culture assay. Yeast isolates and the fungal pathogen were previously grown as described above. An aliquot of 10 μl was streaked on one side of a 9-cm Petri dish (*ϕ* 9 cm) containing PDA at pH 7 (0.5 cm from the edge of the plate). Control treatments were inoculated with 10 μl 0.9% NaCl. A total of four replicates was prepared. Plates were incubated for 24 h at 25°C. After that, a fungal mycelial agar plug (*ϕ* 5 mm) was placed 24 h later at the opposite side of the Petri dish. Plates were incubated for 6 days at 25°C until the fungus on the control plates reached the edge of the plate. After 6 days, the inhibition zone was determined by measuring the area between the fungal hyphae and the yeast colony (area treatment) using ImageJ (FIJI). The inhibition percentage was calculated as


$$\left[\left(\left(\mathrm{area control}\right)-\left(\mathrm{area treatment}\right)\right)*100/\mathrm{area control}\right].$$

The effect of volatile organic compounds (VOCs) on the growth of *F. graminearum* was investigated using two-compartment Petri dishes, which allowed the physical separation between the yeast and the fungal isolates. Yeast and fungal inocula were prepared as described above. An aliquot of 10 μl of the yeast isolates at OD_600nm_ 0.1 was inoculated on one of the compartments of the Petri dish containing PDA (pH 7) and spread evenly. Control treatments were inoculated with 10 μl 0.9% NaCl. A total of four replicates were prepared. Plates were incubated for 24 h at 25°C before inoculation of the fungal pathogen. A mycelium agar plug was placed (*ϕ* 5 mm) on the other compartment of the Petri dish. Plates were sealed three times with plastic wrap and incubated at 25°C for 4 days. Growth inhibition was calculated by measuring the mycelial growth (area treatment) after VOC exposure. The inhibition of the four replicates was calculated by


$$\left[\left(\left(\mathrm{area control}\right)–\left(\mathrm{area treatment}\right)\right)*100/\mathrm{area control}\right].$$

Statistical significance was determined with one-way ANOVA, Tukey’s HSD test (*p* < 0.05).

### Scanning Electron Microscopic Analysis

Scanning electron microscopy (JEOL SEM 6400 equipped with Image Convert for windows) was used to visualize the influence of yeasts on the morphological changes in the fungal hyphae of *F. graminearum*. Samples from the dual culture confrontation assay (described above) were fixed with 1.5% glutaraldehyde in PBS for 1 h while shaking. Then, samples were dehydrated in an increasing percentage of acetone for 20 min each (70, 80, 90, 96, and 100% EtOH) and critical point dried (Baltec CPD-030). Afterward, the samples were sputter coated with platina and palladium to a 20-mm-thickness and stored in a vacuum until use.

## Results

### Isolation and Phylogenetic Delineation of Phyllosphere Yeasts

**Figure 1:**
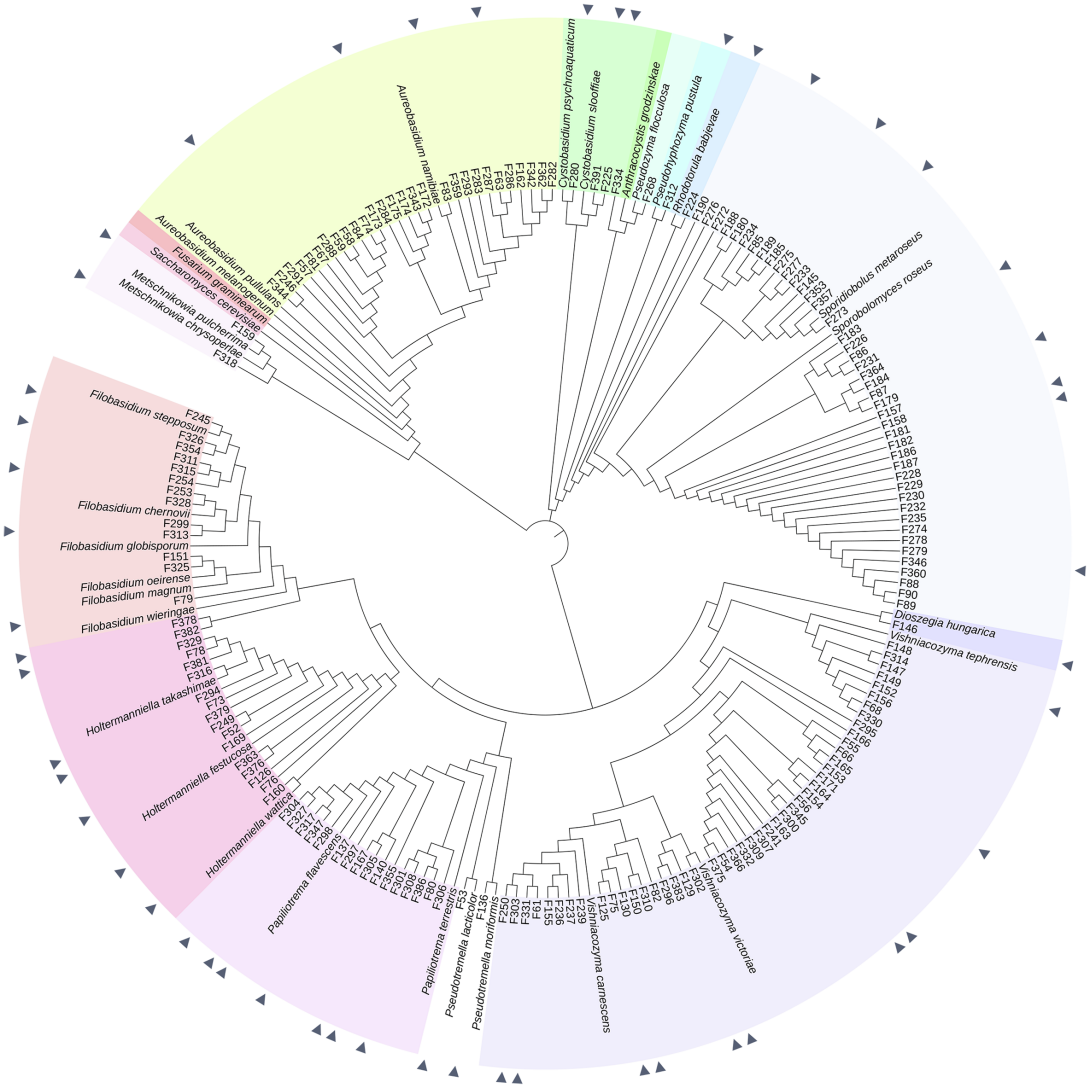
Phylogeny of 175 phyllosphere yeasts isolated from the wheat flag leaf. Phylogeny is based on partial ITS sequences compared to NCBI database. Neighbor Joining tree was constructed with MEGA, MUSCLE for alignment. The iTol software was used to visualize and color-code the tree. Isolates indicated with a triangle are the 51 yeasts selected for the screening of different phenotypic and metabolic traits. *Fusarium graminearum* strain CBS 131786 was used as an outgroup. Type strains used are described in Supplementary Table S1.

**Figure 2:**
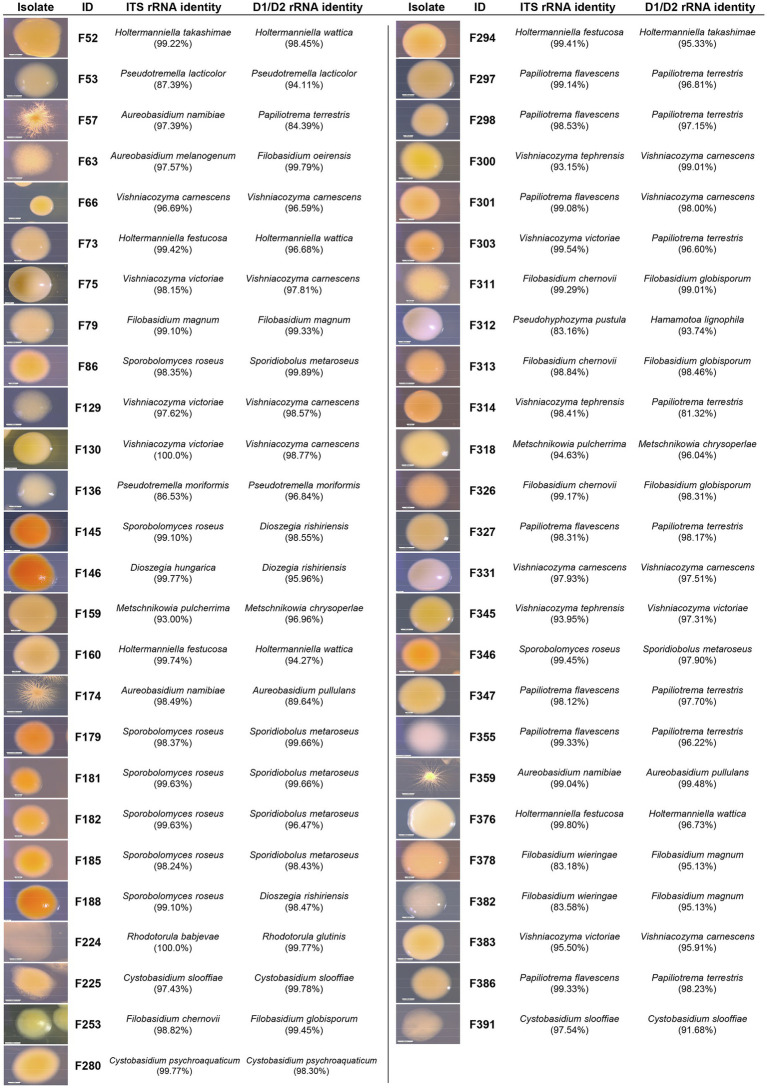
Phenotypic and taxonomic characterization of the selected yeast isolates. Pictures depict 5 to 10-day-old isolates grown on PDA at 25°C. Partial ITS and D1/D2 rRNA gene sequences were compared to the NCBI database for taxonomic identification.

A total of 175 yeasts were isolated from the surfaces and internal tissues of wheat flag leaves. ITS and D1/D2-amplicon sequencing revealed a total of 15 genera, representing 25 different species (Figures 1, 2; Supplementary Table S1). Isolation on SDA and PDA media yielded the highest diversity of isolates, 10 and 9 out of 15 different genera, respectively, followed by YEPD (eight genera). The majority of the yeast isolates belonged to the phylum Basidiomycota (145 isolates; 82.9%), including the genera *Vishniacozyma* (46 isolates; 26.3%), *Sporobolomyces* (42 isolates; 24.0%) and *Papiliotrema* (19 isolates; 9.1%). Less frequent genera detected were *Pseudozyma*, *Anthracocystis*, *Dioszegia,* and *Rhodotorula*. Isolates belonging to the phylum Ascomycota (30 isolates; 17.1%) included *Aureobasidium* (28 isolates; 16.0%) and *Metschnikowia* (2 isolates; 1.1%). For further phenotypic and metabolic characterization, we selected 51 yeast isolates based on their phylogenetic delineation, with at least two isolates of the same genus (Figures 1, 2; Supplementary Figure 1).

### Metabolic Profiling of Phyllosphere Yeasts

**Figure 3:**
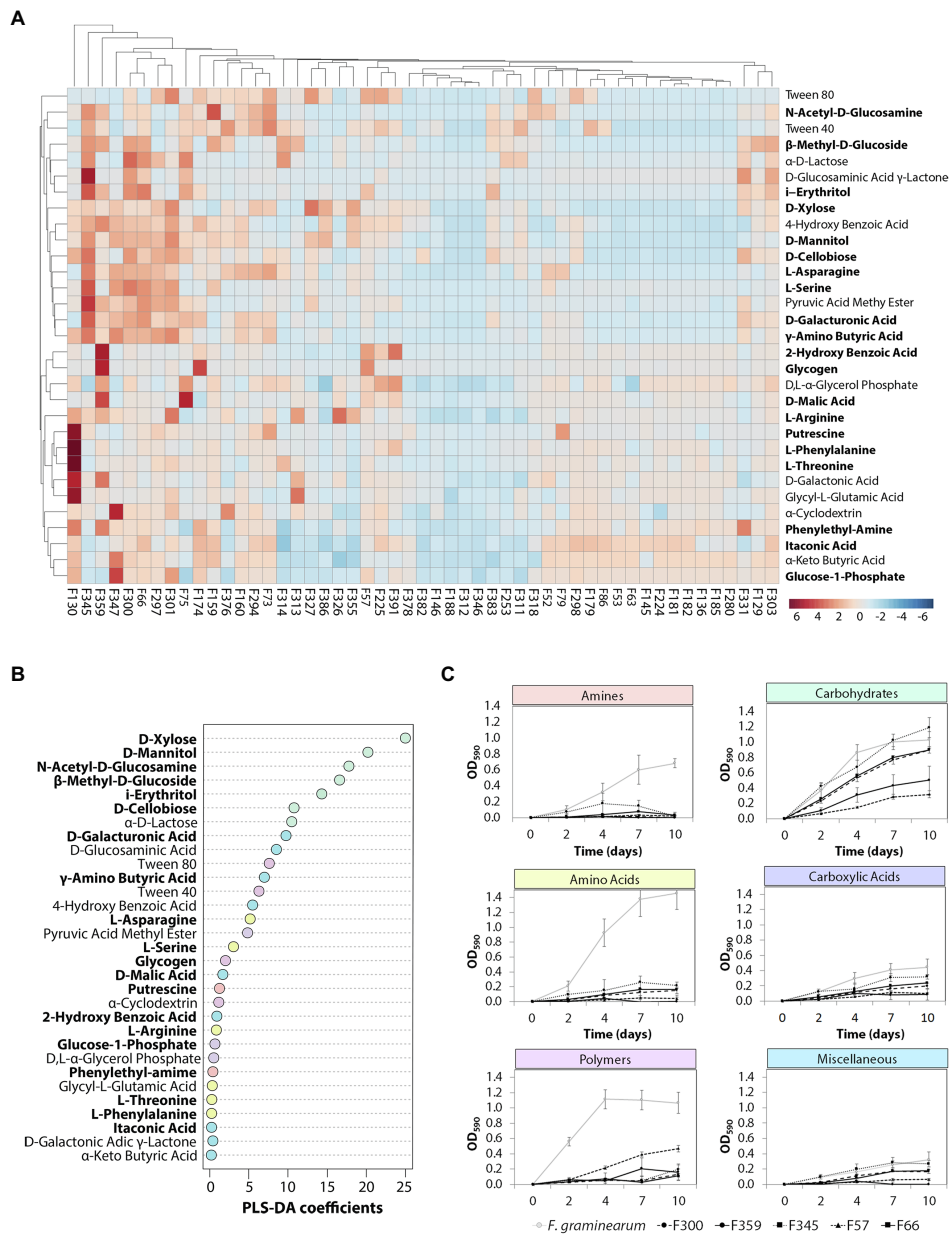
Metabolic profiling of the phyllosphere yeasts. **(A)** Hierarchical cluster and heat-map analyses of nutrient sources utilized by the selected yeast isolates performed with MetaboAnalyst. Yeast isolates were inoculated (OD_600_ = 0.01) in the BIOLOG EcoPlates, incubated for 10 days at 25°C and 180 rpm, growth was measured with a plate reader at OD_590nm_. Columns represent the average of three replicates of each of the 51 isolates. Rows represent the different carbon sources (blue: low abundance, red: high abundance). Compounds of plant and/or microbial origin are displayed in bold. **(B)** Partial least squares-discriminant analysis (PLS-DA) indicating the importance of each carbon source. **(C)** The dynamics of the top 5 isolates including *F. graminearum* with the most diverse metabolic profile per carbon type.

To determine the metabolic diversity of the 51 selected phyllosphere yeasts, Biolog EcoPlate was used to screen for their ability to metabolize 31 nutrient sources of which at least 20 are of plant and/or microbial origin. Growth was measured spectrophotometrically, i.e., average well color development values (Supplementary Table S2). The results showed that isolates classified as *Vishniacozyma* (F300, F345), *Aureobasidium* (F359, F57) or *Papiliotrema* (F301) were able to utilize various carbon sources, while none of the *Sporobolomyces* and *Dioszegia* isolates grew on any of the substrates tested (Figure 3A). More specifically, *Vishniacozyma* isolate F345 had the broadest substrate utilization spectrum, followed by *Vishniacozyma* isolates F75, F300 and F66. Intriguingly, *F. graminearum* strain 8/1 was able to use 24 out of the 31 different carbon sources, indicating a broad substrate utilization spectrum for this prevalent fungal pathogen of wheat leaves. Among the different types of carbon sources tested, carbohydrates were the preferred substrates followed by polymers and carboxylic acid; none of the 51 yeast isolates were able to use amines under the tested conditions. The most frequently metabolized carbohydrates were D-Xylose, D-Mannitol and N-Acetyl-D-Glucosamine, the polymers were Tween 40 and 80, and the carboxylic acids were D-Galacturonic acid and D-Glucosaminic acid (Figures 3B,C).

### Temperature Growth Range of Phyllosphere Yeasts

In the phyllosphere environment, yeasts are exposed to substantial temperature oscillations. To investigate the temperature range for growth, the selected 51 isolates were tested *in vitro* at 4, 10, 25 and 37°C. All yeast isolates were able to grow at 25°C, although the growth rate ranged considerably between the different isolates (Supplementary Table S3; Supplementary Figure 2). Most of the isolates (94%) grew well within 2 days of incubation at 25°C, whereas *Filobasidium* isolate F253 and *Aureobasidium* isolate F63 required up to 7 days of incubation at this temperature. When the temperature was reduced to 10°C, only 27 and 45 isolates were able to grow after 48 and 96 h, respectively. At 4°C, 13 isolates were able to grow after 4 days of incubation, and an additional 31 isolates showed growth with longer incubation. Isolates F225, F280 and F391 classified as *Cystobasidium* were unable to grow at 4°C, but grew at 10°C after 7 days. None of the 51 selected yeast isolates was able to grow at 37°C.

### Biofilm Formation by Phyllosphere Yeasts

Biofilms play a vital role in stress resilience and can protect yeasts against UV radiation and fungicides. Additionally, they can form a physical barrier on leaf surfaces, preventing penetration of the leaf surface by fungal pathogens. *In vitro* biofilm formation was detected by crystal violet staining of liquid cultures after 7 days of growth at 25°C with a threshold of OD_600nm_ at 0.05 to prevent any false positives due to background signal. A total of 19 yeast isolates (38%) were able to form a visible biofilm in at least 2 of the 3 screenings performed (Supplementary Figure 3). Biofilm formation was detected for all *Metschnikowia* (F159 and F318) and *Papiliotrema* (F297, F298, F301, F327, F347, F355, and F386) isolates, whereas no biofilm formation was detected for *Filobasidium, Dioszegia, Rhodotorula* and *Sporobolomyces* isolates under the tested conditions.

### Interactions Between Phyllosphere Yeasts and *Fusarium graminearum*

**Figure 4:**
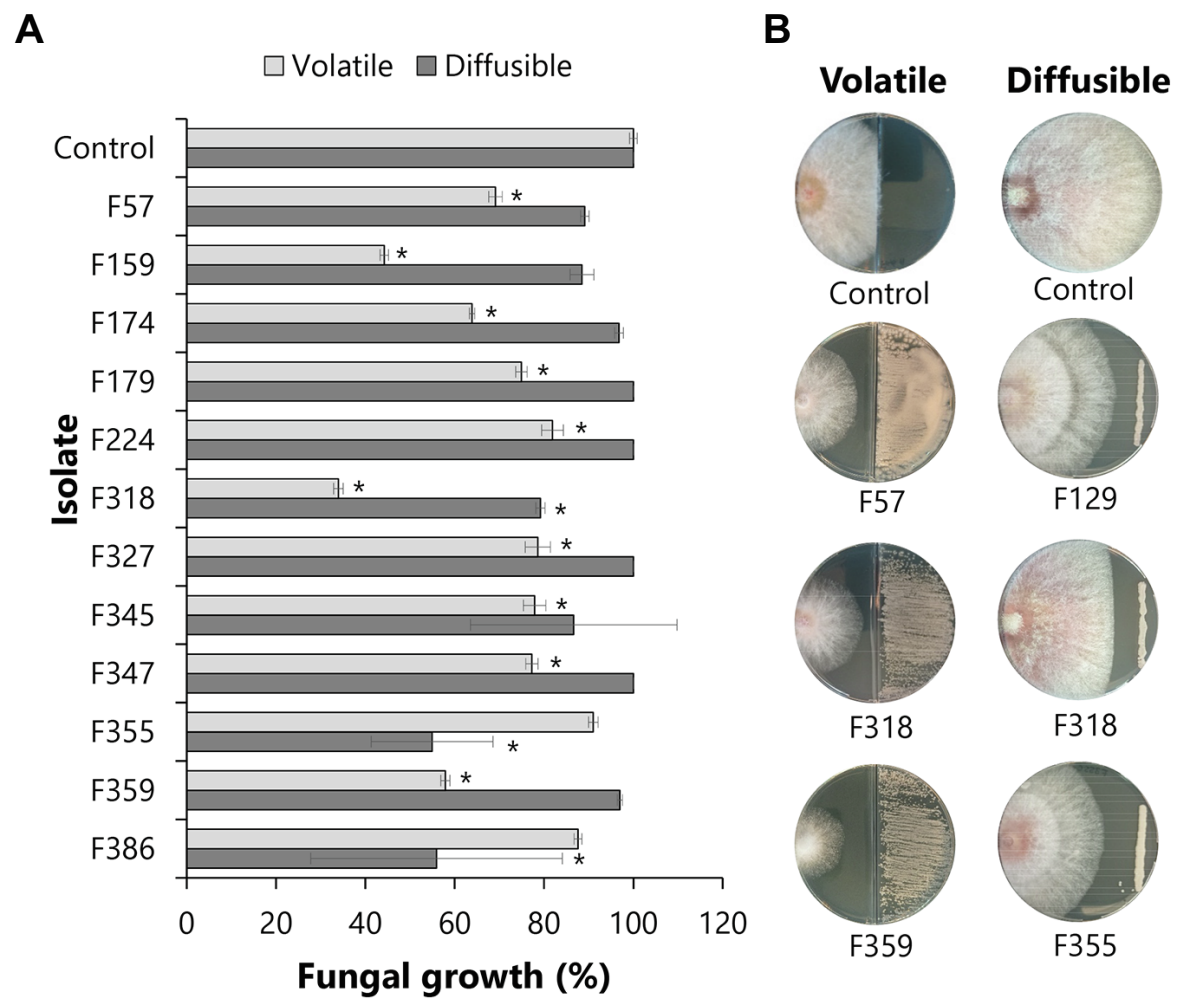
Antagonistic activity of phyllosphere yeasts against *F. graminearum*. Each isolate was screened against *F. graminearum* for the production of volatile organic compounds (VOCs) or agar-diffusible compounds. **(A)** Bars represent the standard deviation of the mean growth percentage of 4 independent replicates. Statistical differences as compared to control (exposed to medium alone) were determined using Student’s *t*-test (*p* < 0.05) and are indicated with an asterisk (*). **(B)** Pictures of the antagonistic activities by representative isolates made after 4 and 6 days of exposure for the volatile and agar-diffusible compounds, respectively.

To investigate the potential of phyllosphere yeasts to inhibit the growth of the leaf pathogen *F. graminearum*, different *in vitro* assays were performed. Dual-confrontation assays showed that several yeast isolates inhibited mycelial growth of the pathogen through the production of volatile and/or diffusible metabolites (Figure 4). Ten of the 51 isolates (19,6%) exhibited significant volatile-mediated antifungal activity. Two of these antagonistic yeast isolates (F318 and F159) that were classified as *Metschnikowia* inhibited the fungal growth by 66 and 56%, respectively. Also isolates classified as *Aureobasidium, Papiliotrema, Rhodotorula*, *Sporobolomyces* or *Vishniacozyma* were able to inhibit the growth of this fungal pathogen *via* volatiles. Two other *Papiliotrema* isolates and one *Metschnikowia* isolate also inhibited hyphal growth *via* agar-diffusible metabolites, by 55, 54 and 21%, respectively. Altogether, these results indicated that various, taxonomically diverse phyllosphere yeasts can inhibit the growth of *F. graminearum via* volatile and diffusible metabolites.

**Figure 5:**
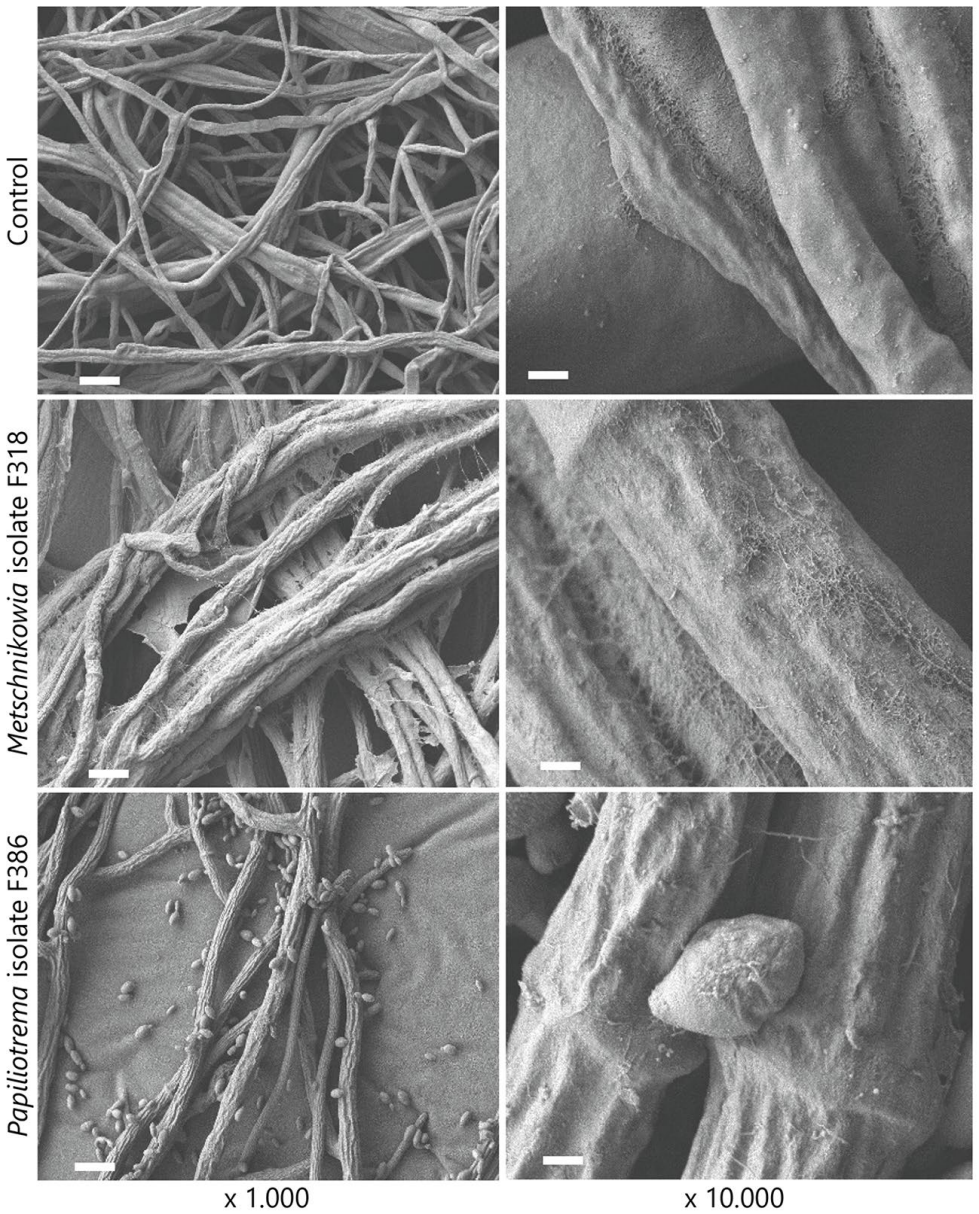
Scanning electron microscopic (SEM) images of the interactions between phyllosphere yeasts and the fungal pathogen *F. graminearum*. Dual culture assays of *F. graminearum* growing alone (top panels) and exposed to *Metschnikowia* (middle panels) and *Papiliotrema* (bottom panels) isolates. Bars represent 10 μm and 1 μm for ×1.000 and ×10.000 magnification images, respectively.

To visualize the interaction between phyllosphere yeasts and *F. graminearum*, scanning electron microscopy showed that *Metschnikowia* isolate F318 and *Papiliotrema* isolate F386 both altered hyphal morphology compared to the control conditions, where smooth fungal hyphae were observed for *F. graminearum* (Figure 5). More specifically, direct confrontation between *Metschnikowia* and *F. graminearum* resulted in the production of an extracellular matrix (presumably extracellular polysaccharides (EPS)) on the hyphal surface as well as in between the hyphae (Figure 5). Confrontation with *Papiliotrema* resulted in shrunken and distorted hyphae (Figure 5).

## Discussion

**Figure 6:**
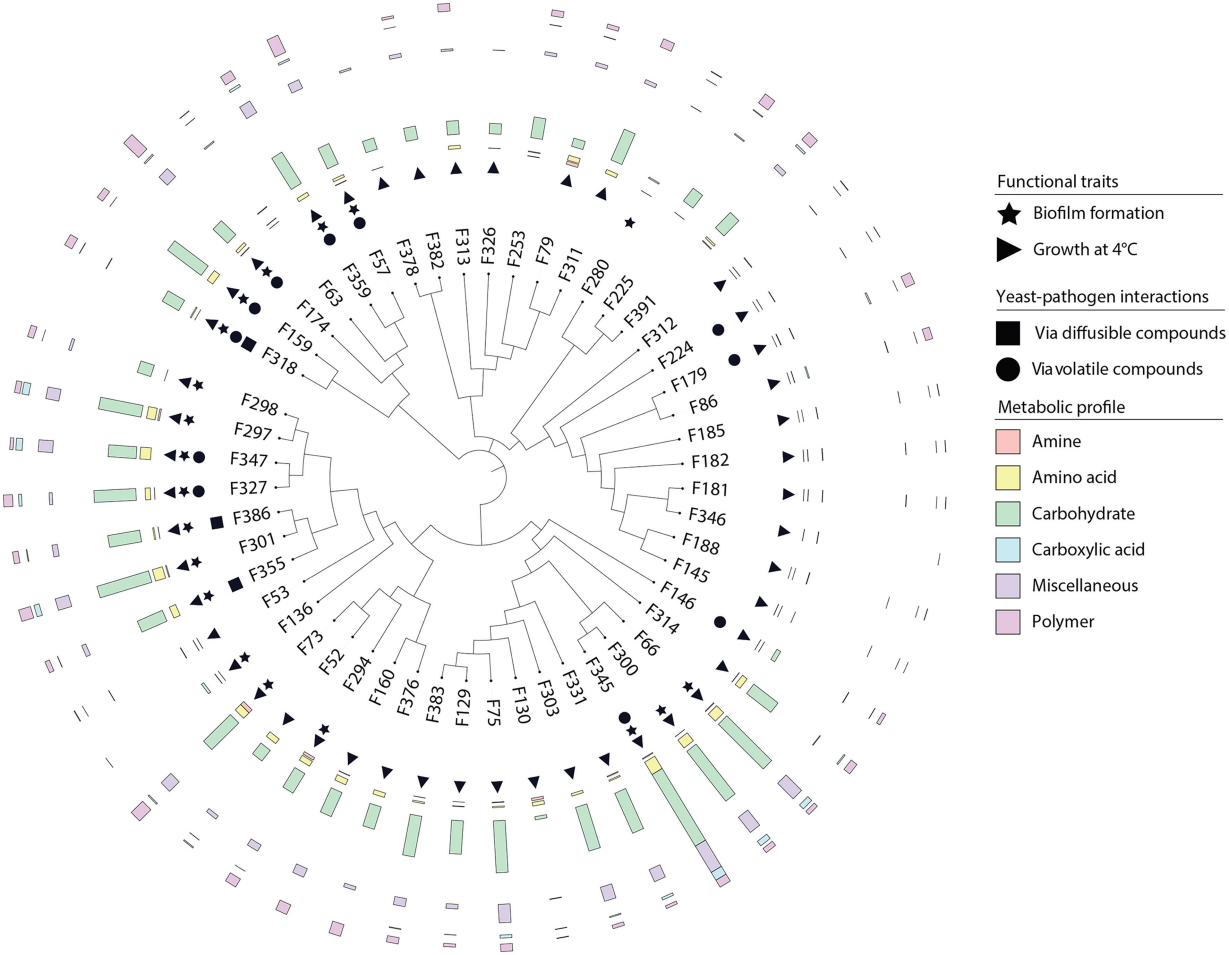
Overview of the adaptive traits of yeasts inhabiting the wheat flag leaf. Neighbor-joining phylogenetic tree based on partial ITS sequences of yeast isolates. Isolates were screened for different traits including biofilm formation, growth at different temperatures, competition with the fungal pathogen *F. graminearum via* the production of agar-diffusible and volatile compounds, and substrate utilization.

Yeasts are well-known for their biotechnological and medical importance, with *Candida* and *Saccharomyces* spp. extensively explored. Plant-associated yeasts have been described for their biocontrol potential, especially in post-harvest disease management but knowledge on yeast ecology and the mechanisms underlying their interactions with other members of the phyllosphere microbiome are still limited. In this study, we examined the diversity of culturable yeasts colonizing wheat flag leaves, their adaptive traits and ability to inhibit the growth of the fungal pathogen *F. graminearum* (Figure 6).

### The Wheat Flag Leaf Harbors a Taxonomically Diverse Reservoir of Yeasts

Previous studies have shown that yeasts colonize a wide range of natural environments, including soils, oceans, insects and plants (Boekhout et al., 2021). Plant-associated yeasts are receiving increased attention in the past two decades, in particular those colonizing fruits (Boekhout et al., 2021). In line with previous culture-dependent and independent studies of the wheat phyllosphere (Karlsson et al., 2014; Sapkota et al., 2015; Knorr et al., 2019), we found that Basidiomycetes were the most frequently isolated yeasts. Among these, yeasts belonging to the genera *Aureobasidium*, *Vishniacozyma* and *Sporobolomyces*, also found in phyllosphere of other crops, such as rice, sugarcane and corn (Nasanit et al., 2015b,c, 2016), were the most abundant. Additionally, we also detected *Pseudohyphozyma* and *Pseudotremella* species, which were described previously for litter and forest soil (Mašínová et al., 2017) but are rarely isolated from the phyllosphere (Elisashvili et al., 2009; Nguyen et al., 2021).

Here we characterized the yeast isolates based on the variable ITS region as well as the 28S domain (also known as D1/D2) for better taxonomic delineation. Yeast taxonomy has long been based on these phylogenetic markers, but the resolution is limited due to the highly conserved sequences in these regions, especially in basidiomycetous yeasts (Li et al., 2020). Therefore, a large number of yeast species are being reclassified (Yurkov et al., 2021). For example, *Cryptococcus* species are being reclassified as *Papiliotrema* and *Filobasidium*, whereas *Candida* species can be found in multiple teleomorphic taxa with different generic names. Hence, the yeasts described here will be subjected in the near future to whole genome sequencing for improved taxonomic delineation.

### Adaptations of Yeasts to the Harsh Phyllosphere Environment

The phyllosphere is a heterogeneous environment where microorganisms are exposed to different (a)biotic stresses. Here we studied a number of traits that may contribute to the yeast’s adaptation to the phyllosphere, including carbon utilization, growth at different temperatures, and biofilm formation. Nutrients, such as carbon compounds, are important for growth and the synthesis of secondary metabolites (Andrews, 1992). A diverse metabolic profile can offer an advantage to withstand the harsh conditions in the phyllosphere, allowing microorganisms to expand their ecological niche by making better use of the available carbon sources (Deak, 2006). Here we showed that the yeasts from the wheat flag leaf displayed diverse metabolic profiles with varying degradation rates. *Vishniacozyma* and *Aureobasidium* isolates, both highly abundant on the wheat flag leaf, displayed the most diverse substrate utilization profiles. Unexpectedly, *Sporobolomyces* isolates were unable to use any of the carbon sources tested under these conditions, despite their frequent and high abundances in wheat leaves found in the present and previous studies (Bashi and Fokkema, 1977; Sapkota et al., 2017). It is likely that *Sporobolomyces* species colonize the wheat flag leaves through the utilization of glucose, fructose or sucrose, sugars that are present in the phyllosphere and enable rapid colonization (Leveau and Lindow, 2000) and competition with leaf pathogens such as *Cochliobolus sativus*, partly by limiting glucose and amino acid availability. Also, *Metschnikowia* isolates have been shown to inhibit the post-harvest pathogen *Colletotrichum gloeosporioides* through competition for glucose and fructose (Tian et al., 2018; Sipiczki, 2020). To begin to understand ecological niche overlap or niche differentiation between the phyllosphere yeasts and the fungal pathogen *F. graminearum* strain 8/1, we also determined the metabolic profile of this pathogen. We found that *F. graminearum* is not only able to utilize most of the nutrient sources tested, but also utilizes amino acids, amines and polymers that could not be utilized by the phyllosphere yeasts tested. This versatile carbon utilization profile likely contributes to the successful infection of a wide range of cereal crops by this pathogen.

In addition to oscillating nutrient availability, phyllosphere yeasts also need to adapt to oscillating temperatures, which can differ drastically between day and night, winter and spring. Yeasts from the genera *Sporobolomyces*, *Rhodotorula* and *Vishniacozyma* have been proposed to tolerate extreme environmental conditions and to help plants adapting to cold environments (Buzzini et al., 2018; Vujanovic, 2021). Our results showed that all isolates from these genera grew at different temperatures, including low temperatures. Also, biofilms play an important role in stress resilience and protection against harsh environmental conditions (Váchová and Palková, 2018), including temperature oscillations. Additionally, biofilms form a physical barrier on injuries of plant surfaces preventing the invasion by pathogenic fungi. We show that biofilm formation was wide-spread among the wheat phyllosphere yeasts, including all *Metschnikowia* and *Papiliotrema* isolates. Previous studies have proposed biofilm formation as one of the antagonistic mechanisms employed by biocontrol *Metschnikowia* isolates on grape wounds (Sipiczki, 2020). This mode of action is hypothesized to be based on growth inhibition of the pathogen by increasing colonization efficiency (e.g., competition for space). However, other biocontrol mechanisms, e.g., production of secondary metabolites, cannot be excluded to contribute to the observed antagonistic activity. Currently, data on biofilm formation by environmental yeasts is sparse (Cordero-Bueso et al., 2017) and our results suggest this may be a more common trait in the phyllosphere. Future investigations involving microscopy should address if these biofilms occur *in planta* and if they contribute to the phyllosphere competence of yeasts.

### Interactions Between Yeasts and the Fungal Pathogen *Fusarium graminearum* in the Phyllosphere

We found that the production of secondary diffusible compounds with inhibitory activity against *F. graminearum* was not a wide-spread mechanism among the yeasts isolated from wheat flag leaves. Only two out of the 51 phyllosphere yeasts tested were able to significantly inhibit hyphal growth. However, it is still possible that these yeast isolates can inhibit the fungal pathogen by competing for nutrients and space, rather than *via* the production of secondary metabolites. Competition for nutrients and space have been proposed as the primary mechanism by which biocontrol yeasts inhibit pathogens (Freimoser et al., 2019). Isolates F355 and F386, which showed the strongest inhibitory effects against *F. graminearum*, are closely related to *Papiliotrema flavescens*, formerly known as *Cryptococcus flavescens/C. nodaensis*. Earlier studies on *P. flavescens* OH182.9, isolated from wheat heads, showed no antagonism in *in vitro* experiments, but this isolate was able to reduce disease incidence caused by *F. graminearum* by 56% in a bioassay (Khan et al., 2001). Field experiments where different winter wheat cultivars were inoculated with this strain also demonstrated reduced disease severity of *F. graminearum* by 60% (Schisler et al., 2002b; Khan et al., 2004).

Scanning electron microscopy further showed hyphal morphological changes on the hyphae of *F. graminearum* during the interaction with specific yeast isolates. The interaction with *P. flavescens* (isolate F386) not only reduced hyphal growth but also changed the hyphal morphology from smooth to shrunken and distorted. This morphological change has been previously observed for *F. graminearum* confronted by different *Paenibacillus peoriae* strains, bacteria with a high potential of biocontrol and plant growth promotion (Ali et al., 2021). Interestingly, the interaction of *F. graminearum* with *Metschnikowia* isolate F318, an isolate displaying little reduction of hyphal growth *in vitro*, led to considerable changes in hyphal morphology. These results suggest that *Metschnikowia* isolate F318 imposed stress to the fungal pathogen resulting in the production of extracellular polysaccharides (EPS). Biofilm formation by other *Fusarium* species, more specifically *F. oxysporum*, has been described to protect the fungal pathogen against several fungicides (Peiqian et al., 2014).

Contrary to diffusible secondary metabolites, volatile compounds with inhibitory effects on *F. gramineraum* were more frequently produced by the phyllosphere yeasts. These low molecular weight compounds serve as a means of interspecies communication from a distance and are proposed as an effective biological control strategy against multiple pathogens (Tilocca et al., 2020). The most promising volatile-producing antagonists belonged to *M. pulcherrima* (isolates F159 and F318), *Aureobasidium pullulans* (isolates F57, F174 and F359) and *Papiliotrema flavescens* (isolates F327 and F347), inhibiting hyphal growth by up to 65%. Volatile compounds produced by *Metschnikowia* and *Aureobasidium* species have been previously described for their biocontrol potential against several fungal pathogens, including *A. alternata* and *B. cinerea* (Oro et al., 2018). The volatile compounds ethanol, 2-phenylethanol and ethyl acetate have been proposed to be involved in this inhibitory activity (Oro et al., 2018; Contarino et al., 2019; Sipiczki, 2020; Yalage Don et al., 2020) and hypothesized to disrupt the fungal membranes leading to leakage and deformed hyphae (Yalage Don et al., 2020). To date, however, most of the knowledge on yeast volatiles stems from studies using yeasts of biotechnological importance, e.g., the production of volatiles by yeasts and their effects on wine aroma (Bordet et al., 2020). Further research efforts should be put on characterization of the volatile compounds produced by environmental yeasts and their ecological roles.

## Concluding Remarks

The wheat flag leaf is a diverse reservoir of yeasts which can withstand the harsh conditions found in the phyllosphere through different mechanisms. Here we provide a first insight into the genetic and phenotypic diversity of yeasts living on and in the wheat flag leaf. Further research should focus on the molecular mechanisms underlying the interactions between yeasts, including the identification of genes and metabolites involved in antifungal activity. To this end, current approaches and tools already in use for model yeasts can be employed for comparative genomics and functional analysis of the phyllosphere yeasts. Unraveling the mechanisms underlying the antagonistic activities would not only improve our understanding of the ecology of phyllosphere yeasts, but also contribute to the discovery of novel yeast-based biocontrol products.

## Data Availability Statement

Data was deposited at the ENA database under accession number PRJEB51687 (secondary accession ERP136341), available from June 19th, 2022.

## Author Contributions

LG and VC designed the study, processed and interpreted the data, and drafted the manuscript. LG, CV, and VC performed the experiments. All authors contributed to the article and approved the submitted version.

## Funding

All authors are supported by the Novo Nordisk Foundation (Grant NNF19SA0059348).

## Conflict of Interest

The authors declare that the research was conducted in the absence of any commercial or financial relationships that could be construed as a potential conflict of interest.

## Publisher’s Note

All claims expressed in this article are solely those of the authors and do not necessarily represent those of their affiliated organizations, or those of the publisher, the editors and the reviewers. Any product that may be evaluated in this article, or claim that may be made by its manufacturer, is not guaranteed or endorsed by the publisher.

## Footnotes

^1^ https://www.ncbi.nlm.nih.gov/
