# Supplementary material for: Genetic, Phenotypic and Metabolic Diversity of Yeasts From Wheat Flag Leaves
Source: Front Plant Sci. 2022 Jul 7;13:908628. doi: 10.3389/fpls.2022.908628 (PMC9301128; doi:10.3389/fpls.2022.908628)
Supplement: Supplementary file 1 [file Data_Sheet_1.PDF]

Supplementary Material

Genetic, phenotypic and metabolic diversity of yeasts from wheat flag leaves

Linda Gouka[1], Caroline Vogels[1], Lars Hestbjerg Hansen[2], Jos M. Raaijmakers[1,3], Viviane Cordovez[1*]

***Correspondence:** Viviane Cordovez, v.cordovez@nioo.knaw.nl

Supplementary Figure 1. Phylogeny of 51 phyllosphere yeast isolates selected for the screening of different phenotypic and metabolic traits. Phylogeny is based on D1/D2 domains of the large subunit of the 26S rRNA gene sequences compared to NCBI database. Neighbor Joining tree was constructed with MEGA, MUSCLE for alignment.

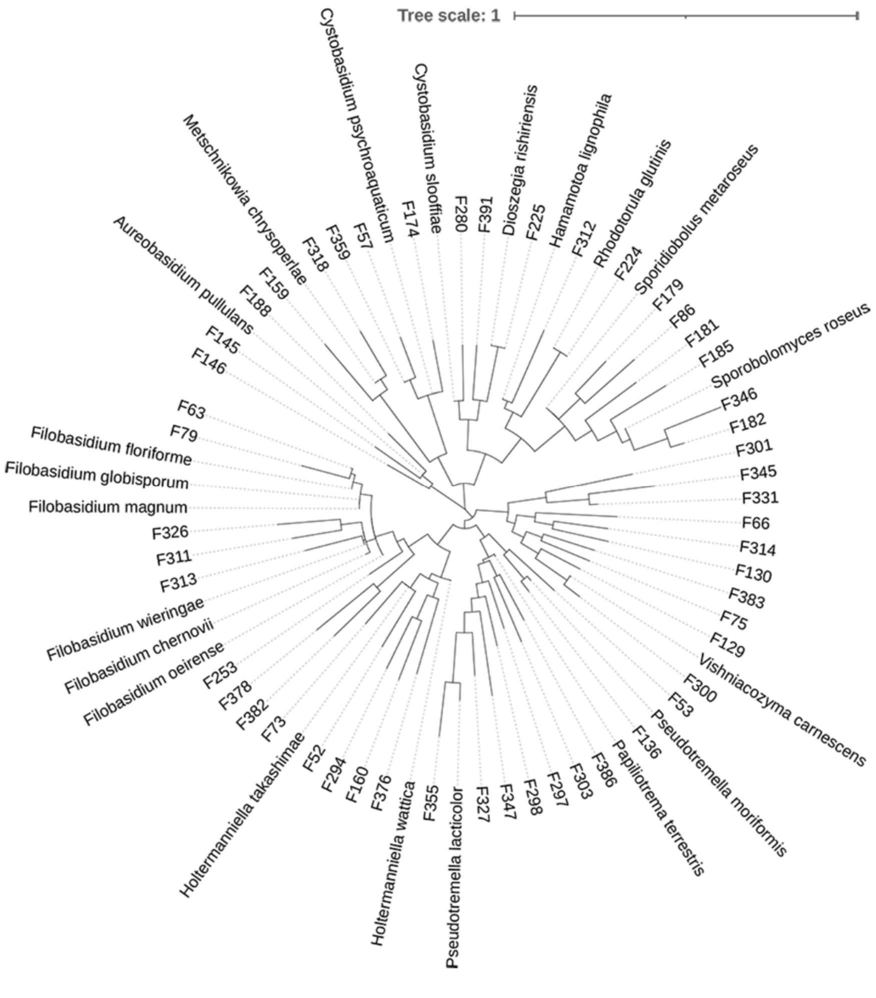

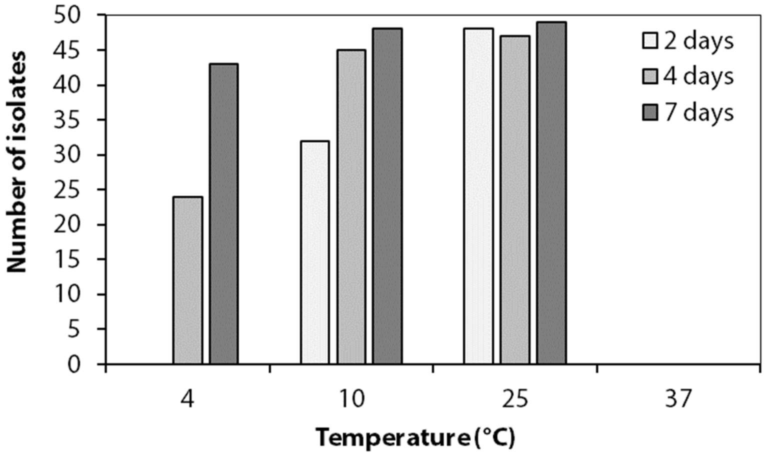

Supplementary Figure 2. Growth of phyllosphere yeasts at different temperatures. Bars represent the number of phyllosphere yeasts able to grow in the different temperature range. Additional information can be found in Supplementary Table S3.

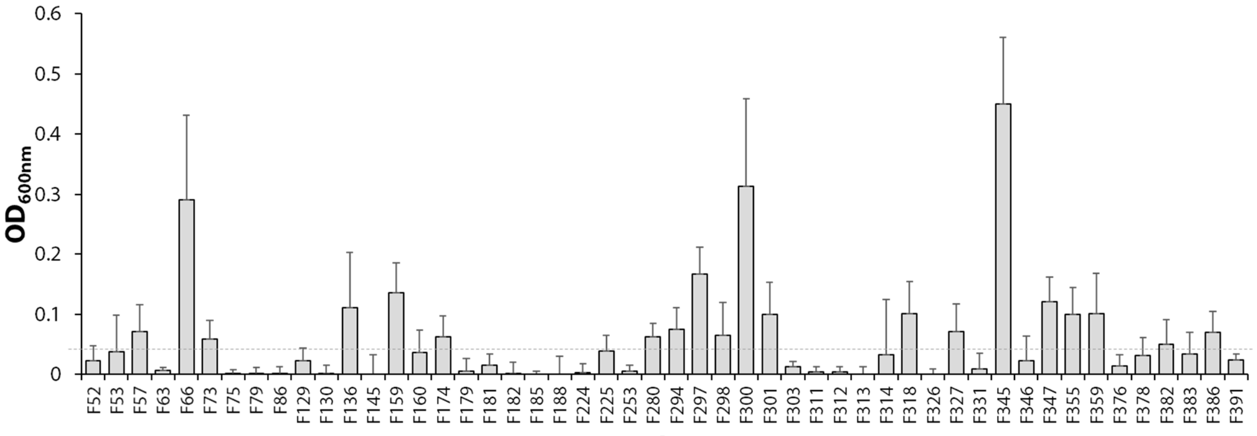

Supplementary Figure 3. Biofilm formation by phyllosphere yeasts. Optical density (OD_{600nm}) of cells in biofilm was determined after 7 days. Bars present the standard deviation of the means of 4 replicates and at least 2 independent experiments. Isolates displaying an optical density higher than 0.05 were considered to form biofilm.
